# Molecular and morphological characterization of *Paurodontella composticola* n. sp. (Nematoda: Hexatylina, Sphaerulariidae) from Iran

**DOI:** 10.21307/jofnem-2019-034

**Published:** 2019-06-06

**Authors:** Mehrab Esmaeili, Ramin Heydari, Ahmad Kheiri, Weimin Ye

**Affiliations:** 1 Department of Plant Protection, College of Agriculture and natural resources, University of Tehran, Karaj, Iran; 2 Nematode Assay Section, North Carolina Department of Agriculture and Consumer Services, Agronomic Division, Raleigh, NC, 27607, USA

**Keywords:** 28S D2/D3, Molecular phylogeny, Morphology, New species, Taxonomy

## Abstract

A new species of the genus *Paurodontella*, *P. composticola* n. sp., collected from Nazar Abad City, Alborz Province, Iran, is described and illustrated. The new species has a body length of 803–1053 μm (females *n* = 10) and 620 and 739 μm (males *n* = 2). The cuticle is weakly annulated with four lateral lines. Cephalic region is annulated and continuous with body contour. The stylet is 8.0 to 9.0 μm long with asymmetrical knobs. Esophageal basal bulb is present with a small posterior extension projecting into the intestine. Excretory pore is situated at the level of esophageal basal bulb region. Post-uterine sac is 5 to 8 μm long and uterus is without diverticulum. Tails of both sexes are similar, short and sub-cylindrical. Males have 24 to 25 μm long bursa leptoderan and spicules 24 or 25 µm long. A non-branching oviduct is present to form a uterine diverticulum; the new species is closely related to five known species of the genus, namely *P*. *asymmetrica*, *P*. *balochistanica*, *P*. *densa*, *P*. *iranica* and *P*. *niger*. It most closely resembles *P*. *iranica*, but differs from it morphologically by a shorter stem-like extension projecting into lumen of intestine and male with sub-cylindrical tail vs conoid. In addition to morphological comparisons, the molecular phylogenetic analyses based on 733 bp of the partial sequence of 28S D2/D3 expansion segments of the large subunit rDNA gene (LSU) revealed this as a new species.

The genus *Paurodontella* (Husain and Khan, 1968) is a diverse and worldwide distributed Paurodontid taxon. To date, *Paurodontella* contains 14 nominal species (Handoo et al., 2010; Esmaeili et al., 2016b; Yaghoubi et al., 2018), including *P. aberrans* (Nandakumar and Khera, 1969; Sumenkova, 1975), *P. apitica* (Thorne, 1941; Husain and Khan, 1968), *P. asymmetricus* (Tikyani and Khera, 1968; Sumenkova, 1975), *P. densa* (Thorne, 1941; Husain and Khan, 1968), *P. minuta* (Husain and Khan, 1968), *P. niger* (Thorne, 1941; Husain and Khan, 1968), *P. sohaili* (Maqbool, 1982), *P. auriculata* (Anderson, 1985), *P. balochistanica* (Handoo et al., 2010), *P. myceliophaga* (Handoo et al., 2010), *P. iranica* (Golhasan et al., 2016), *P. persica* (Esmaeili et al., 2017a), *P. parapitica* (Esmaeili et al., 2016b), and *P. gilanica* (Yaghoubi et al., 2018). Among these, *P. iranica*, *P. persica*, *P. gilanica* and *P. parapitica* were described from Iran.

*Paurodontella* is mainly characterized by the presence of basal stylet knobs, the location of excretory pore near the nerve ring, the esophageal bulb being present with long/or short stem-like extension, the absence of post uterine sac (PUS) or present only in rudimentary form, simple vulval lips and bursa not being leptoderan (Siddiqi, 2000). According to the classification by Siddiqi (2000), *Paurodontella* belongs to the subfamily Paurodontinae (Thorne, 1941), family Paurodontidae (Thorne, 1941), superfamily Sphaerularioidea (Lubbock, 1861), suborder Hexatylina (Siddiqi, 1980) in the order of Tylenchida (Thorne, 1949). Siddiqi (2000) considered the family Paurodontidae a junior synonym of Sphaerulariidae, since the genera included in these families are morphologically similar and have similar life cycles. However, there is not enough available molecular data to support these statements. Biologically, in Paurodontidae, a fungus-feeding generation is well known and nothing is known about entomoparasitic forms, whereas in Sphaerulariidae, an entomoparasitic form is present. Some other nematologists (Chizhov, 2004; Andrássy, 2007; Handoo et al., 2010) followed Siddiqi’s opinion considering Paurodontidae as a synonym of Sphaerulariidae. In this study, Siddiqi’s (2000) scheme was followed.

A history of the taxonomic studies on members of the superfamily Sphaerularioidea and a list of species occurring in Iran were given by Esmaeili et al. (2016b). In order to study the species diversity of this superfamily in Iran, we conducted several samplings in cultivated and natural areas of Iran during August 2017 and, as a result, a population of a *Paurodontella* species has been found in compost from a few places where mushrooms failed to grow. This population morphologically resembled a group of *Paurodontella* species by having a short stylet with asymmetrical knobs at the base, the subventral knobs larger than dorsal, a short stem-like extension projecting and extended immediately behind the base of esophageal basal bulb, short PUS and four incisures in the lateral fields. These traits prompted us to perform much detailed morphological and molecular study to compare with all previously described species. These observations revealed that this species appeared to be morphologically and morphometrically distinct from any existing *Paurodontella* species. Thus, the objective of this study was to characterize morphologically and molecularly a population of the genus *Paurodontella* that is described herein as *P*. *composticola* n. sp.

## Material and methods

### Sampling, extraction, mounting, and drawing

Specimens of *Paurodontella composticola* n. sp. were obtained from compost collected in Nazar Abad City, Alborz Province, Iran in August 2017. To obtain a cleaner suspension of nematodes, the tray of extraction was employed (Whitehead and Hemming, 1965). Specimens for light microscopy were killed by gentle heat, fixed in a solution of 4% formaldehyde + 2% glycerol and transferred to anhydrous glycerin, according to De Grisse (1969), and mounted on permanent slides. Specimens were examined using an Olympus BH-2 (Japan) compound microscope at magnifications up to 1,000× magnification. Measurements were carried out using a drawing tube attached to a Nikon E200 light microscope (Japan). All measurements were expressed in micrometers (μm) and photographs of nematodes were taken by a digital camera attached with the same microscope.

### DNA extraction, PCR, and sequencing

Nematode DNA was extracted from single live individuals. Single nematode specimen was transferred to an Eppendorf tube containing 16 µl ddH_2_O, 2 µl 10× PCR buffer and 2 µl proteinase K (600 µg/ml) (Promega, Benelux, The Netherlands) and crushed for 2 min with a micro-homogeniser, Vibro Mixer (Zürich, Switzerland). The tubes were incubated at 65°C for 1 hr, then at 95°C for 10 min. In total, 1 µl of extracted DNA was transferred to an Eppendorf tube containing 2.5 µl 10× NH_4_ reaction buffer, 0.75 µl MgCl_2_ (50 mM), 0.25 µl dNTPs mixture (10 mM each), 0.75 µl of each primer (10 mM), 0.2 µl BIOTAQ DNA Polymerase (BIOLINE, UK) and ddH_2_O with a final volume of 25 µl. The 28 S D2/D3 was amplified using forward primer D2A (5′-ACA AGT ACC GTG AGG GAA AGT TG-3′) and reverse primer D3B (5′-TCG GAA GGA ACC AGC TAC TA-3′) (Nunn, 1992).

PCR cycle conditions were as follows: one cycle at 94°C for 2 min, followed by 35 cycles of denaturation at 94°C for 30 s, annealing temperature of 55°C for 45 s, extension at 72°C for 3 min, and finally one cycle at 72°C for 10 min. PCR products were purified after amplification using ExoSAP-IT (Affmetrix, USB products), quantified using a Nanodrop spectrophotometer (Nanodrop Technologies, Wilmington, DE, USA) and used for direct sequencing in both directions using the same PCR primers. The resulting products were purified and run on a DNA multicapillary sequencer (Model 3130XL genetic analyser; Applied Biosystems, Foster City, CA, USA), using the BigDye Terminator Sequencing Kit v.3.1 (Applied Biosystems, Foster City, CA, USA), at the Stab Vida sequencing facilities (Caparica, Portugal). The newly obtained sequence was submitted to the GenBank database under accession number MH427864 for partial 28S D2/D3 rDNA.

### Phylogenetic analyses

The molecular sequence of *Purodontella composticola* n. sp. was compared with those of other nematode species available in GenBank using the BLAST homology search program. Our newly obtained DNA sequence was edited with ChromasPro1.5 2003-2009 (Technelysium Pty Ltd, Helensvale, Australia) and aligned using ClustalW (http://workbench.sdsc.edu; Bioinformatics and Computational Biology group, Dept. Bioengineering, UC San Diego, CA). All available species of *Paurodontella* and some other Hexatylina species from GenBank were also selected for phylogenetic analysis (Table 1). Outgroup taxon was chosen according to previously published data (Esmaeili et al., 2016b). The base substitution model for the sequences data was evaluated using MODELTEST version 3.06 (Posada and Criandall, 1998) based on the Akaike-supported model (Arnold, 2010). Bayesian analysis was performed to confirm the tree topology for 28S D2/D3 rDNA gene using MrBayes 3.1.0 (Huelsenbeck and Ronquist, 2001), running the chain for 1,000,000 generations and setting the “burnin” at 1,000. Markov Chain Monte Carlo method was used within a Bayesian framework to estimate the posterior probabilities of the phylogenetic trees (Larget and Simon, 1999) using the 50% majority rule. The *λ* ^2^ test for homogeneity of base frequencies and phylogenetic tree was performed using PAUP* version 4.0 (Sinauer Associates, Inc. Publishers, Sunderland, MA).

**Table 1.** Species used for the analysis of phylogenetic relationships and the accession numbers for LSU sequences deposited in GenBank Species.

	Accession number	Authors	Species	Accession number	Authors
*Abursanema iranicum*	KF885742	Yaghoubi *et al*. (2014)	*Howardulla* sp.	JX291131	Chizhov *et al*. (2012)
*Anguina* sp.	KU317615	unpublished data	*Litylenchus coprosma*	GU727547	Zhao *et al*. (2012)
*Anguina tritici*	DQ328723, KC818620	Subbotin *et al*. (2006)	*Nothotylenchus persicus*	KT149799	Esmaeili *et al*. (2015)
Anguinata	KY067443	unpublished data	*Nothotylenchus phoneixae*	KX549319	Esmaeili *et al*. (2017c)
*Cephalenchus* sp.	KU723245	Pereira and Baldwin (2016)	*Nothotylenchus* sp.	MH243749	unpublished data
*Cephalenchus cephalodiscus*	KX685166	Pereira *et al*. (2017)	*Paurodontella composticolla* n. sp.	MH427864	Present study
*Cephalenchus daisuce*	KX462033	Pereira *et al*. (2017)	*Paurodontella gilanica*	MF543010	Yaghoubi *et al*. (2018)
*Cephalenchus nemoralis*	KU723245	Pereira and Baldwin (2016)	*Paurodontella iranica*	KP642168	Golhasan *et al*. (2016)
*Deladenus siricidicola*	AY633444	Ye *et al*. (2007)	*Paurodontella parapitica*	KU522237	Esmaeili *et al*. (2016b)
*Deladenus* sp.	JX104332	unpublished data	*Paurodontella persica*	KP000034	Esmaeili *et al*. (2017a)
*Deladenus* sp.	JX104322	Morris *et al*. (2013)	*Paurodontoides* sp.	MG836264	Esmaeili *et al*. (2018)
*Ditylenchus arachis*	KX426052, KX426054	Wang *et al*. (2017)	*Rubzovinema* sp.	KF155283	Koshel *et al*. (2014)
*Ditylenchus destructor*	EU400626	Wang *et al*. (2017)	*Rubzovinema* sp.	KF373736	Koshel *et al*. (2014)
*Ditylenchus dipsaci*	FJ707363, KT806479, JF327763, JF327765,	Douda *et al*. (2013)	*Sphaerularia cf. bombi*	AB733664, AB733665, DQ328726	Ye *et al*. (2007)
*Ditylenchus gigas*	HQ219217, KC310734	Vovlas *et al*. (2011)	*Sphaerularia vespae*	AB300596	Ye et al. (2007)
*Ditylenchus oncogenus*	KF612015	Vovlas *et al*. (2016)	Sphaerularioidea	KR920361	unpublished data
*Ditylenchus persicus*	KX463285	Esmaeili *et al*. (2016a)	*Subanguina moxae*	JN885540	Yao *et al*. (2012)
*Ditylenchus stenurus*	KX400577	Esmaeili *et al*. (2017b)	*Subanguina radicicola*	JN885539	Yao *et al*. (2012)
*Ditylenchus weischeri*	MG551886	Madani and Tenuta (2017)	*Subanguina* sp.	KT205566, KT205560, KX776479	unpublished data
*Fergusobia* sp.	AY633446	Ye et al. (2007)	*Travassosinema claudiae*	KX844645	Morffe and Hasegawa (2017)
*Tylenchida* sp.	FJ661086	unpublished data	*Travassosinema* sp.	LC214839	unpublished data
*Tylenchomorpha* sp.	LC147027	unpublished data	–	–	–

## Results

**Figure 1:**
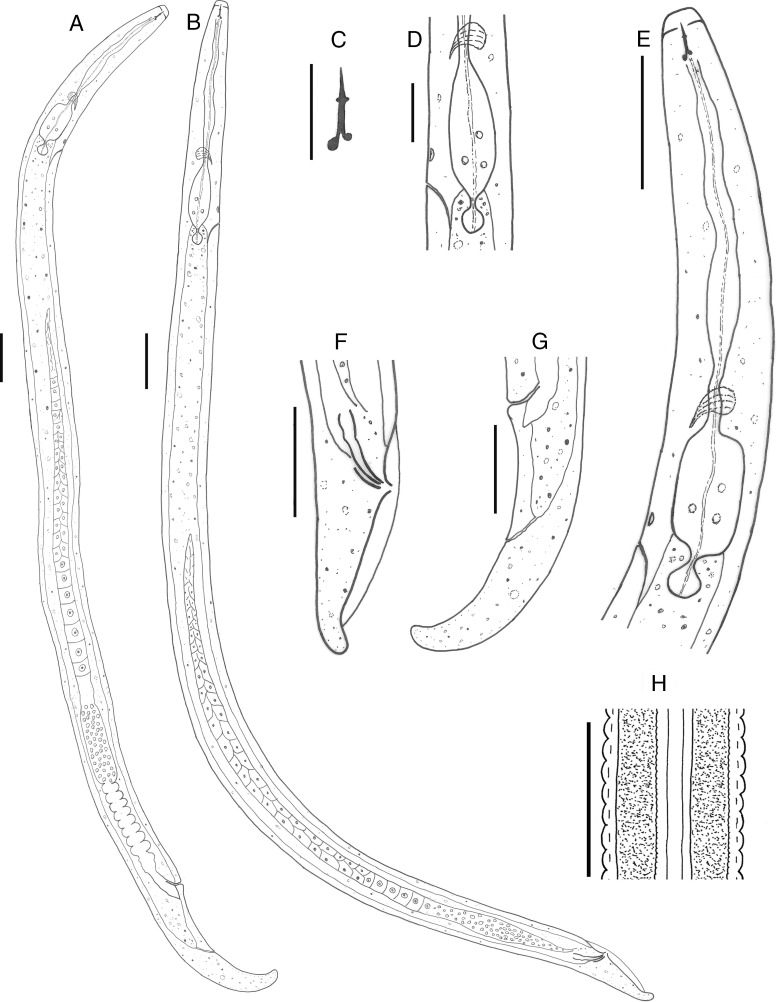
Line drawing of *Paurodontella composticola* n. sp. (A): female entire body; (B): male entire body; (C): stylet; (D): medium bulb region of female; (E): female esophageal region; (F): male posterior body; (G): female posterior region; (H): lateral field of female. (All scale bars 30 μm, except for C = 10 μm; D = 20 μm).

**Figure 2:**
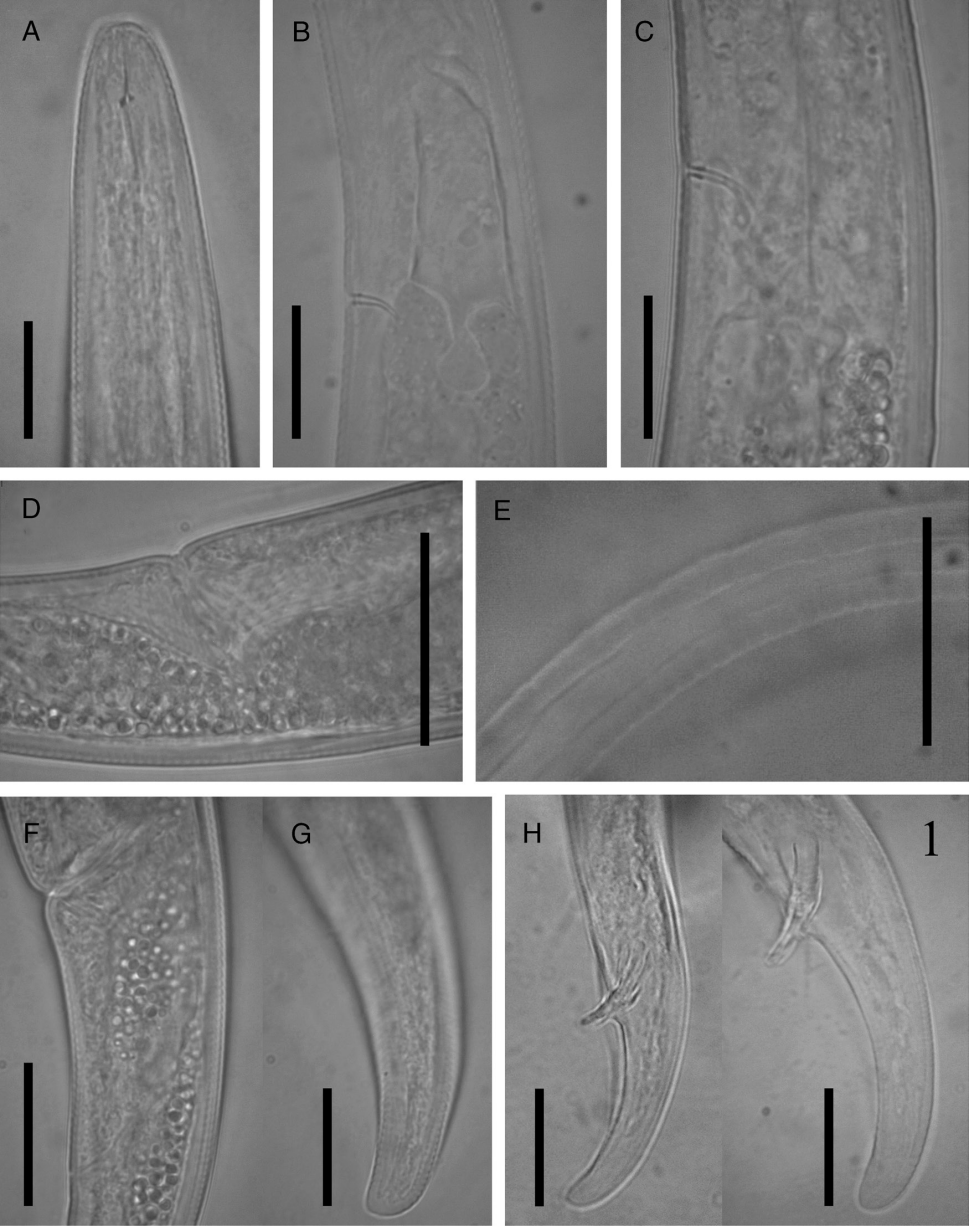
Photomicrographs of *Paurodontella composticola* n. sp. (A): female anterior end; (B, C): basal bulb region of female; (D): vulval region; (E): lateral field of female; (F): vulva to anus distance; (G): female posterior region; (H, I): male posterior body. (All scale bars 20 μm).

**Figure 3:**
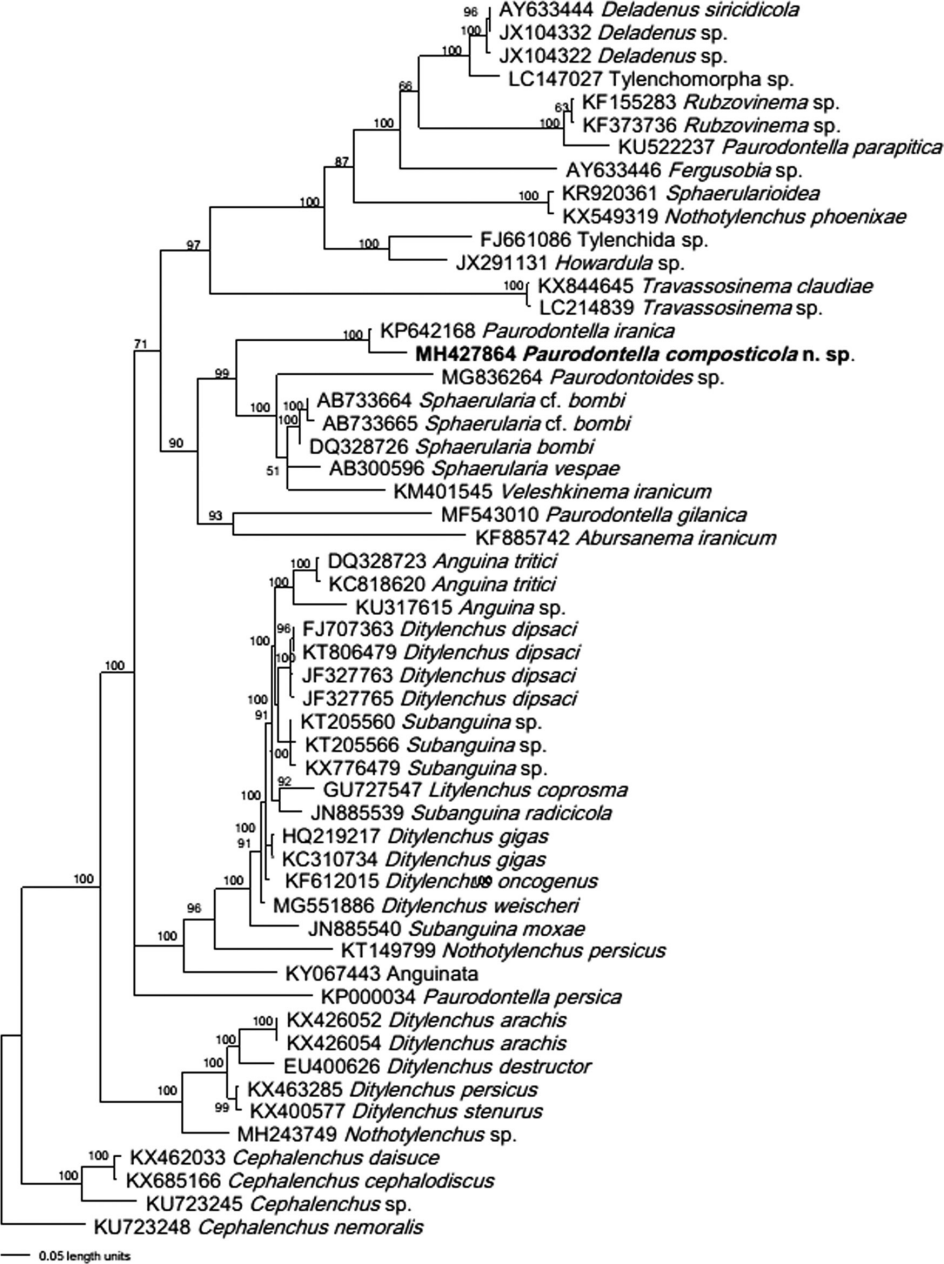
Bayesian consensus tree inferred from 28S D2/D3 rDNA under TVM + I + G model (−lnL = 13,024.7812; AIC = 26,067.5625; freqA = 0.1584; freqC = 0.2128; freqG = 0.3327; freqT = 0.2961; *R*(a) = 1.0917; *R*(b) = 5.2109; *R*(c) = 2.5802; *R*(d) = 0.8631; *R*(e) = 5.2109; *R*(f) = 1; Pinva = 0.1293; Shape = 0.8648). Posterior probability values exceeding 50% are given on appropriate clades. The new sequence is in boldface.

*Paurodontella composticola* n. sp.

(Table 2; Figs. 1–3)

**Table 2.** Morphometrics of *Paurodontella composticola* n. sp.

	Female		Male
Character	Holotype	Paratypes	Paratypes
*n*	–	10	2
L	1,005	940 ± 95.9 (803–1,053)	620–793
a	24.5	25.9 ± 4.2 (18.6–31.5)	23.0–30.1
b	6.7	5.1 ± 0.6 (5.4–6.9)	4.2–5.1
c	22.3	24.5 ± 4.4 (17.8–32.1)	20.7–22.5
c'	2.0	2.1 ± 0.4 (1.5–2.5)	1.8–2.4
*V* or *T*	89.5	90.0 ± 1.2 (78.4–91.2)	40.6–50.0
Lip region height	3	2.9 ± 0.2 (2.5–3.0)	3.0
Lip region width	8.5	6.6 ± 0.1 (6.0–7.0)	7.0–8.0
Stylet length	9	8.8 ± 0.5 (8.0–9.0)	8.0–8.5
Nerve ring from anterior end	85	91.3 ± 12.0 (79–103)	85–93
E. pore from anterior end	136	129.6 ± 10.2 (116–144)	92–124
Pharynx length	150	154.9 ± 16.7 (136–182)	140–156
PUS length	5	5.6 ± 1.2 (5.0–8.0)	–
Ovary length or testis	624	585 ± 80.8 (408–656)	300–310
Body diameter at vulva (VBD)	33	29.4 ± 5.7 (23–40)	–
Vulva to anus	57	52.8 ± 3.8 (46–57)	–
Anal (cloacal) body diam.	22	19 ± 2.8 (16–24)	14–17
Tail length	45	39.3 ± 6.0 (26–45)	30–33
Spicules length (arc line)	–	–	24–25
Gubernaculum length	–	–	7.0–8.0
Bursa (% of tail)	–	–	96

Note: All measurements in μm and in the form: mean ± s.d. (range).

### Description

#### Female

They are free-living. The body is slender or semi-obese, slightly ventrally curved after fixation. Cuticle is finely annulated, 1.3 to 2.2 μm wide at mid-body. The lateral field is marked by four incisures; outer incisures strongly crenate and wider, inner weakly crenate. Cephalic region is wide, rounded, annulated and continuous with body contour, 2.5 to 3.0 μm high, 6.0 to 7.0 μm wide. Labial plate is flattened. Stylet is delicate with asymmetrical knobs, with the subventral knobs being larger than the dorsal. Stylet length is almost equal to the head width; conus is occupying 36 to 46% of the total stylet length. Deirids are present at the level of excretory pore. The dorsal gland orifice is 1.5 to 2.5 μm posterior to stylet knobs. Esophageal corpus is a cylindrical tube with a long and fusiform median bulb, without valvular apparatus; isthmus is cylindrical and slender, encircled by nerve ring. The excretory pore is located at the level of the basal esophageal bulb region, located at 116 to 144 μm far from anterior end. Esophageal basal bulb is present with small posterior extension, 6.0 to 8.0 μm long, projecting into the intestine. Hemizonoids are distinct, situated 5.0 to 7.0 μm anterior to the excretory pore. Vulva is near anus as a transverse slit, posterior, *V* = 87 to 91%. Reproductive system is prodelphic, with an outstretched gonad. Vagina is muscular, directed forward, and 8 to 9 μm long. Crustaformeria is composed of 8 to 10 rows. The oviduct is non-branching so as to form a uterine diverticulum. Spermatheca is spherical with round sperm cells 2.2 to 3.2 μm in diam.; in young females, it is empty. Oocytes are present in a single and two rows at the proximal end. Ovary is reflexed at the tip in some individuals, in young females, it is outstretched. A short PUS is present. The tail is short, sub-cylindrical with broadly rounded tip, ventrally curved. Phasmids are not observed.

#### Male

They are rarely found (ratio of 1:5 females). They are very similar to females in general morphology, except for the reproductive system. Cuticle is present with fine annulation, about 1.5 to 1.8 μm apart. The tail is conical, with rounded tip without mucron, arcuate after fixation. Testis is single, outstretched. Spermatocytes are arranged in double rows. Spicule is cephalate, ventrally arcuate. Gubernaculum is simple. Bursa is annulated, leptoderan.

#### Entomoparasitic generation

Not found.

### Type host and locality

Females and males are found in compost from a few places where mushrooms failed to grow in Nazar Abad City, Alborz Province, Iran, during August 2017. (GPS coordinates: 35°48 N, 51°00E, 1,380 m a.s.l.).

### Type material

Holotype female, two paratype females and two paratype males (Slides PCS001 and PCS002) were deposited in Nematode Collection of Department of Plant Protection, College of Agricultural and Natural Resources, University of Tehran, Karaj, Iran. Two female paratypes were deposited in National Nematode Collection of the Department of Nematology, Iranian Research Institute of Plant protection, Tehran, Iran. Two paratype females were deposited in USDA Nematode Collection, Beltsville, MD, USA.

### Etymology

The species epithet refers to Latin name of compost, the origin of species, where the type specimens were collected.

## Diagnosis and relationships

*Paurodontella composticola* n. sp. is an amphimictic species characterized by the following characters: a large body length of 803 to 1,053 μm (females) and 620,739 μm (males), lateral fields marked by four incisures, stylet 8 to 9 μm long, stylet with minute and asymmetrical basal knobs, a short stem-like extension projecting into lumen of intestine, excretory pore being located at the level of esophageal basal bulb region *ca* 116 to 144 μm from anterior end in females, the absence of a uterine diverticulum branch, *V* = 87 to 91, the presence of a short PUS, sub-cylindrical tail, rare male with 24 to 25 μm long spicules and leptoderan bursa.

According to Handoo et al. (2010), species of *Paurodontella* can be divided into those with/without a uterine diverticulum. Due to the presence of a relatively long body, short PUS, shape of posterior body and a non-branching oviduct to form uterine diverticulum, the new species *P. composticola* n. sp. is closely related to five known species in the genus: *P. asymmetrica*, *P. balochistanica*, *P. densa*, *P. iranica* and *P. niger*.

Compared to *Paurodontella asymmetrica*, the new species has a shorter stylet, 8 to 9 vs 13 to 14 μm, a longer body length, 803 to 1,053 vs 330 to 380 μm, a greater a-ratio, 18.6 to 31.5 vs 16 to 19, a sub-cylindrical tail vs conical tail with pointed end, a higher c-ratio, 17.8 to 32 vs 10 to 11, more posteriorly located vulva, *V* = 87.5 to 91.5 vs 81 to 83, and presence of male in population vs absence. It differs from *P*. *balochistanica* by having a shorter stylet, 8.0 to 9.0 vs 10 to 11 μm, with asymmetrical knobs vs symmetrical, a lower a-ratio, 18.6 to 31.5 vs 33 to 34, lateral field with four incisures wherein outer incisures crenate and inner smooth vs lateral field with four smooth incisures, sub-cylindrical tail vs conoid tail with a terminal mucron, higher c-ratio, 17.8 to 32 vs 17.1 to 17.8, more posteriorly located vulva, *V* = 87.5 to 91.5 vs 88.0 to 88.6, and presence of males vs absence. It differs from *P. densa* by having a longer body, 803 to 1053 vs 400 μm, labial plate flattened vs rounded, PUS present vs absent, lateral field with four vs six lines, posteriorly located vulva, *V* = 87.5 to 91.5 vs 82, sub-cylindrical tail vs subacute terminus. It differs from *P. niger* by having a longer body, 803 to 1053 vs 400 μm, lateral field with four incisures vs six incisures, more posteriorly located vulva, *V* = 87.5 to 91.5 vs 80, and sub-cylindrical tail vs conical with pointed terminus tail. The new species most closely resembles *P*. *iranica* but differs from it by having an esophageal basal bulb with a short stem-like extension, 6.0 to 8.0 μm, vs a long stem-like extension, 16 to 35 μm, into the lumen of intestine and male tail sub-cylindrical vs conical with rounded tip.

Due to the presence of non-valvate median esophageal bulb and basal bulb with an extension projecting into the intestine, *Paurodontella* is close to other members of the family Paurodontidae, including species of genera *Abursanema* (Yaghoubi et al., 2014), *Bealius* (Massey and Hinds, 1970), *Misticius* (Massey, 1967), *Paurodontus* (Thorne, 1941), and *Paurodontoides* (Jairajpuri and Siddiqi, 1969).

*Paurodontella composticola* n. sp. can be distinguished from *Abursanema* by having a stylet with basal knobs (vs stylet without knobs) and the presence of bursa (vs absence or rudimentary). The new species differs from *Bealius* by having an esophageal basal bulb with a stem-like extension projecting into the intestine ventrally (vs dorsally) and leptoderan bursa vs peloderan. If differs from *Misticius* by having four incisures in lateral field (vs absence of lateral field) and excretory pore opening near the basal bulb level (vs near the stylet base). Genus *Paurodontella* can be distinguished from *Paurodontus* by having the stylet with asymmetrical basal knobs (vs asymmetrical), and by the presence of a rudimentary PUS (vs prominent). It can also be distinguished from *Paurodontoides* by having a rudimentary PUS (vs prominent) and having a leptoderan (vs peloderan) bursa.

### Molecular phylogeny

The amplification of the partial 28S D2/D3 rDNA gene sequence from *Paurodontella composticola* n. sp. specimens yielded a single fragment of approximately 800 bp based on gel size.

To determine the phylogenetic relationships of *Paurodontella composticola* n. sp. with other nematode species, an edited long partial sequence of 733 bp of 28S rDNA with accession number MH427864 was used. The BLAST search revealed that the sequence is unique with no highly matched sequence available in GenBank; only 93% identity was achieved with *P. iranica* with accession number of KP642168. When the new species was compared for the 28S D2/D3 region of *P*. *iranica*, they shared 677 identical nucleotides (93.01%), 27 insertions/deletions (3%) and 1 indel over 731 total characters. The phylogenetic tree generated from 28S D2/D3 alignment by BI analysis under the TVM+I+G model is presented in Figure 3, which contained 53 in-groups and one outgroup taxon. This tree rooted with *Cephalenchus nemoralis* (Mizukubo and Minagawa, 1985) (KU723248) showed *P. composticola* n. sp. in a monophyletic clade with *P. iranica* (KP642168) with 100% support. This clade is close to a highly supported (99%) monophyletic clade, including species of the subfamily Paurodontinae (*Paurodontoides* and *Paurodontella*) and Sphaerulariinae (*Abursanema* and *Sphaerularia*), revealing the polyphyly of these genera based on limited species available in GenBank. This is supported by the morphological similarities of these taxa, mostly in the shape of the esophagus (e.g. non-valvate or non-muscular median bulb). *Paurodontella persica* is not in a monophyletic clade, which revealed that their morphological similarity is convergence in evolution and they are not a monophyletic group. According to Siddiqi (2000), Paurodontidae is a family dubia, clearly implying a possible synonymy with Sphaerulariidae. However, the fact that the grouping of *Sphaerularia* (Sphaerulariidae) and the new species (as a member of Paurodontidae) were clustered together with 70% of posterior probability, our phylogenetic study based on partial 28S D2/D3 appears to support this opinion. This finding is supported by previous studies as well (Yaghoubi et al., 2014; Miraeiz et al., 2015; Esmaeili et al., 2016b, 2017a; Mobasseri et al., 2017).

## Discussion

*Paurodontella* spp. share some contrasting characters that vary in the species, e.g. asymmetrical/symmetrical stylet knobs, the presence/absence of a deep vertical amphidial aperture covered by an auriform cuticular flap, a chamber-like structure surrounding the esophageal bulb/or simple, a short/long basal esophageal bulb stem attaching/projecting into the intestine, an oviduct branching to form an uterine diverticulum or not, PUS present, rudimentary, or absent, and leptoderan bursa (Siddiqi, 2000; Handoo et al., 2010). Handoo et al. (2010) documented a diagnostic key for identification of the species of the genus: the presence/or absence of PUS and a uterine diverticulum in the oviduct branching are the most important characters for species delimitation under this genus. Yaghoubi et al. (2018) listed the morphological characters for species delimitation within *Paurodontella* and closely related genus *Paurodontus*. They argued that PUS (longer in *Paurodontus* as corresponding body diam.) and basal pharyngeal bulb extension (shorter in *Paurodontus*) are the main morphological characters to separate these genera. *Paurodontella composticola* n. sp. is unique within the genus by having a stylet with asymmetrical knobs, a short esophageal basal bulb extension and a uterine diverticulum through a non-branching oviduct. However, the importance of these features for separating species in *Paurodontella* has to be reinforced by molecular studies on more species of the genus.

The superfamily Sphaerularioidea comprises a diverse group of taxa that are separated from each other on the basis of their morphological and/or biological characters. Besides, using the currently available sequences of representatives of several genera/species of this group, members of the families do not form monophyletic groups based on the partial 28S D2/D3 rDNA sequence. In our present 28S tree, members of Sphaerularioidea have occupied separate clades within the phylogenetic tree. For example, the subfamilies of Sphaerulariidae (Paurodontinae and Sphaerulariinae) are in separate clades, distantly related to each other. This polyphyly, regarding how the new species and current species of *Paurodontella* are clustered in this study, is also seen with several genus such *Abursanema* (Paurodontinae), *Deladenus* (Thorne, 1941) (Neotylenchinae), *Howardula* (Cobb, 1921) (Allantonematinae), *Rubzovinema* (Slobodyanyuk, 1991) (Rubzovinematinae), *Veleshkinema* (Miraeiz et al., 2015) (Sphaerulariinae) (Figure 3). Likewise, Koshel et al. (2014) using SSU-ITS1-5.8S-LSU rDNA sequences documented the polyphyletic condition of families, such as Anguinidae Nicoll, 1935 (1926), Allantonematidae (Pereira, 1931) and Neotylenchidae (Thorne, 1941).

In conclusion, due to the very limited number of Hexatylina species molecularly characterized, the relationships among subfamilies Paurodontinae and Sphaerulariinae are still not clearly resolved, despite of the sister relationship of members. Further evidence using valuable morphological diagnostic characters and molecular studies with different markers is needed to confirm.
